# Validation of a rapid one-step high sensitivity real-time quantitative PCR system for detecting major *BCR*-*ABL1* mRNA on an International Scale

**DOI:** 10.1186/s40064-016-2258-6

**Published:** 2016-05-10

**Authors:** Chikashi Yoshida, Hirohisa Nakamae, Linda Fletcher, Daisuke Koga, Takayuki Sogabe, Itaru Matsumura, Yuzuru Kanakura, Susan Branford, Tomoki Naoe

**Affiliations:** Department of Hematology, National Hospital Organization Mito Medical Center, 280, Sakuranosato, Ibarakimachi, Higashiibarakigun, Ibaraki, 311-3193 Japan; Hematology, Department of Internal Medicine, Osaka City University Graduate School of Medicine, Osaka, Japan; Department of Genetic and Molecular Pathology, Centre for Cancer Biology, SA Pathology, Adelaide, Australia; Diagnostic Division, Otsuka Pharmaceutical, Tokushima, Japan; Hematology, Department of Internal Medicine, Kinki University School of Medicine, Osaka, Japan; Hematology and Oncology, Osaka University Graduate School of Medicine, Osaka, Japan; School of Pharmacy and Medical Science, University of South Australia, Adelaide, Australia; School of Medicine, University of Adelaide, Adelaide, Australia; School of Molecular and Biomedical Science, University of Adelaide, Adelaide, Australia; National Hospital Organization Nagoya Medical Center, Nagoya, Japan

**Keywords:** Chronic myeloid leukemia, *BCR*-*ABL1*, Real-time quantitative PCR, International Scale, Conversion factor

## Abstract

**Background:**

Detection and quantitation of *BCR*-*ABL1* transcripts are crucial for managing patients with chronic myeloid leukemia (CML). Although real-time quantitative polymerase chain reaction (RT-qPCR) can be measured on an International Scale (IS), this has not become fully universal. By using a WHO international standard panel established for calibrating secondary standards based on the IS, we have previously developed an RT-qPCR kit, ODK-1201, for quantification of major *BCR*-*ABL1*.

**Results:**

In this study, the reliability of kit-specific conversion factor 1.12 was validated by exchanging patients’ samples between three local clinical laboratories and a reference laboratory. The mean bias of the local method after IS conversion was 1.6 fold lower than the reference method. The clinically-useful sensitivity of the kit was further evaluated for monitoring patients with deep molecular response. Based on the correlation of the IS values between ODK-1201 and the reference laboratory method, the detection level of the kit was estimated as 0.0032 % *BCR*-*ABL1*^IS^.

**Conclusions:**

ODK-1201 is a highly sensitive one-step RT-qPCR system for detecting *BCR*-*ABL1* on the IS in 2 h after RNA extraction, thus contributing to standardization of molecular monitoring in CML.

## Background

BCR-ABL is a constitutively active tyrosine kinase generated by the Philadelphia chromosome translocation, and is recognized as the underlying pathogenetic mechanisms of chronic myeloid leukemia (CML) (Yoshida and Melo 2004). Tyrosine kinase inhibitors (TKI) have dramatically changed the prognosis of patients with chronic phase CML, enabling high overall survival (O’Brien et al. 2003). They also allow a proportion of patients to achieve major molecular response (MMR), which is associated with a high progression-free survival, as firstly reported for the International Randomized Study of Interferon and STI571 (IRIS) trial (Hughes et al. 2003). Recent studies have indicated that achievement of early molecular response is translated into desirable long term prognosis (Hughes et al. 2014; Jabbour et al. 2014; Marin et al. 2012). Furthermore, cessation of TKI treatment has been attempted in patients who achieved deep molecular response, and has succeeded in maintaining prolonged treatment-free remission in a subset of patients (Mahon et al. 2010; Ross et al. 2013). Therefore, accuracy and standardization of molecular monitoring are essential for the management of CML patients.

Such monitoring is usually done by measuring *BCR*-*ABL1* mRNA using real-time quantitative polymerase chain reaction (RT-qPCR) (Branford et al. 1999; Mensink et al. 1998). However, the data obtained by RT-qPCR demonstrate inter-laboratory variability, which hampers the proposal of guidelines for clinicians and the comparison of results in clinical studies (Zhang et al. 2007). For the harmonization of molecular monitoring in CML, an International Scale (IS) was proposed in 2005 and widely used in current clinical recommendations and guidelines (Baccarani et al. 2013; Hughes et al. 2006; NCCN 2016). It is based on a standardized baseline defined as 100 % *BCR*-*ABL1*^IS^, and a MMR defined as 0.1 % *BCR*-*ABL1*^IS^ (i.e., a 3-log reduction from the standardized baseline, also referred to as MR3), as established in the IRIS trial (Hughes et al. 2006). To use this system, each laboratory needs to calibrate their data to the IS by multiplying the result of their measurement by a laboratory-specific conversion factor (CF) (Branford et al. 2008). However, it is not-straightforward to obtain the CF because it requires the exchange of clinical samples between local and reference laboratories. To solve the issue, primary BCR-ABL standard material based on the IS was developed and approved by the WHO (White et al. 2010).

We have previously reported the development of a one-step RT-qPCR kit, ODK-1201. The kit-specific CF was 1.12 determined by using the WHO standard panel. The assay performance was shown as limit of detection 0.0007 % using diluted samples containing low level of *BCR*-*ABL1*, but not using clinical samples (Nakamae et al. 2015). This is an extension of the work aimed to validate the kit-specific CF by patient’s sample exchange between local laboratories and a worldwide reference laboratory, which has been recognized as a standard method for adaptation to the IS. Furthermore, it is intended to verify accuracy, inter-laboratory reproducibility, and clinically-useful sensitivity for improved clinical utility of the kit.

## Methods

### Study design and data analyses

Two clinical studies (ODK-1201-01, ODK-1201-02) were carried out to identify of the performance of ODK-1201 and to get the laboratory-specific CF, sponsored by Otsuka Pharmaceutical Co. Ltd. Total 468 CML patients were recruited for these studies, and peripheral blood samples were taken after written informed consent in 24 institutions in Japan, between July 2012 and October 2013. These studies were conducted in accordance with the Declaration of Helsinki, and its design was approved by each hospital’s Institutional Review Board. The samples were firstly analyzed in three commercial laboratories, BML, Inc., SRL, and LSI Medience Corporation (previously Mitsubishi Chemical Medience Corporation) in Japan, and thereafter in SA Pathology, Adelaide, Australia, a recognized reference laboratory (Branford et al. 2008; Hughes et al. 2003). Only samples with the standard b2a2 (e13a2) and/or b3a2 (e14a2) *BCR*-*ABL1* transcripts were included. For the validation of obtained values by ODK-1201, negative samples and samples with >10 % *BCR*-*ABL1*^IS^ were excluded from analysis, since the IS measurement was shown to be effective in the range of 10 % *BCR*-*ABL1*^IS^ or below (Branford et al. 2008). A patient bias conversion method was used to determine the CF for the IS, as previously described (Bland and Altman 1986; Branford et al. 2008). In the validation step, the bias between the kit and the reference method was calculated before and after conversion to the IS using the specific CF of each method. The estimated mean bias of the kit method after conversion was calculated as the average fold difference compared with the reference method.

### Experimental design

A 7-mL blood sample was taken from the patients in participating hospitals and transferred to the local laboratories. RNA extraction was carried out using the QIAamp RNA Blood Mini Kit (QIAGEN, Duesseldorf, Germany) in BML, RNeasy Mini Kit (QIAGEN) in SRL, or RNeasy Lipid Tissue Mini Kit (QIAGEN, Duesseldorf, Germany) in LSI, respectively. The study diagnostic kit in this study was coded ODK-1201. It is based on a HawkZ05 Fast One-Step RT-qPCR Kit (Roche, Indianapolis, IN, USA). In this kit, reverse transcription and quantitative PCR are performed in one tube. The analysis was carried out on an ABI™ 7500 Fast Dx system (Applied Biosystems, Foster City, California). In the quantitation with this kit, specific regions of major *BCR*-*ABL1* mRNA and *ABL1* mRNA transcripts were amplified by RT-qPCR and quantitated by using probes specific to the amplified products. The primers for *ABL1* hybridize to not only *ABL1* but also *BCR*-*ABL1*. In each assay with ODK-1201, 1 μg of RNA (10 μL) was added to a mixture of 13 μL of mix solution (R1) for RT-qPCR and 2 μL of Mn(OCOCH3)_2_ solution and applied to ABI™ 7500 Fast Dx system. The assay conditions have been described previously (Nakamae et al. 2015). Five 20-fold dilutions from a standard RNA solution included in the kit were used to create the standard curve for *BCR*-*ABL1*and *ABL1*. The test results were compared to the standard curve to yield the quantity of *BCR*-*ABL1* and *ABL1* mRNA. To calculate the kit-specific CF for the IS, the WHO International Standard panel was obtained from The National Institute for Biological Standards and Control (NIBSC, UK), and analyzed according to the suggested method for calibration of secondary standards (http://www.nibsc.org/documents/ifu/09-138.pdf). The panel comprises four ampoules, each containing freeze-dried *BCR*-*ABL1*-positive and -negative cells at various ratios from 0.01 to 10 % *BCR*-*ABL1*^IS^. RNAs were isolated from each ampoule with a QIAamp RNA Blood Mini Kit (QIAGEN) and were subjected to the measurement of major *BCR*-*ABL1* and *ABL1* mRNAs with the ODK-1201 kit. The CF of the ODK-1201 was calculated by comparing the values of % *BCR*-*ABL1/ABL1* obtained with the kit with those from the WHO standard panel. The RT-qPCR method carried out at the Adelaide reference laboratory has been previously detailed (Branford et al. 1999).

## Results

**Fig. 1:**
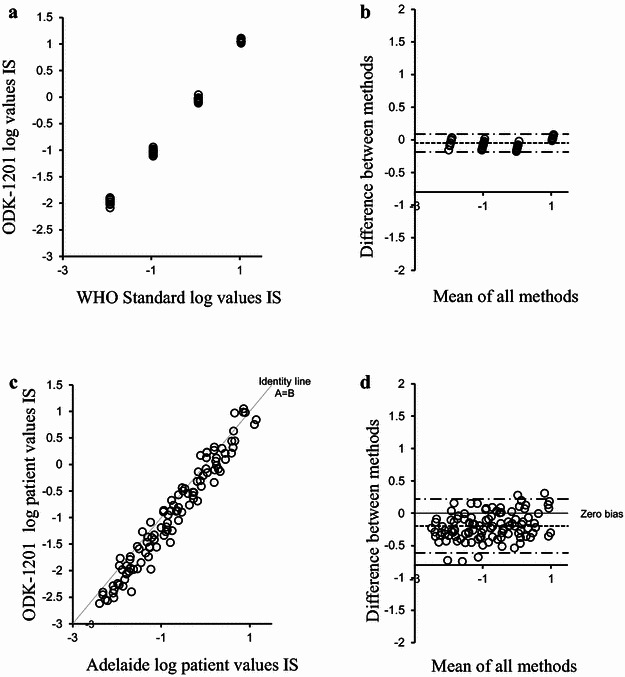
CF calculation and validation of ODK-1201. **a**, Visual inspection of the data used for the CF calculation for ODK-1201 using WHO standard material. **b** Data *bias plot* using the Bland and Altman method (Bland and Altman 1986). **c** Visual inspection of the converted validation data of the kit-specific CF. **d** Validation data *bias plot* using the Bland and Altman method (Bland and Altman 1986)

The ODK-1201 kit-specific CF was 1.12 determined by using the WHO standard panel by following the method for calibration of secondary standards as outlined in White et al. (2010). The calculation method of the CF was presented in Fig. 1a, b, which was not detailed in our previous report (Nakamae et al. 2015).

The reliability of the kit-specific CF was next validated by analyzing the patients’ samples. A total of 106 samples were first tested with ODK-1201 in three local clinical laboratories (36, 34, and 36 samples each), and then sent to the reference laboratory. None of the samples was degraded. The *BCR*-*ABL1* values from the reference and the local laboratories were converted to the IS by multiplying by their specific CFs. The samples with positive *BCR*-*ABL1* values were converted to the IS using the CF 1.12 and compared (Fig. 1c, d). The mean bias of the local method after IS conversion was 1.6 fold lower than the reference method data. The upper and lower 95 % limits of agreement after IS conversion was −0.614 fold and 0.220 fold, respectively. The mean bias in the three individual local laboratories after IS conversion was 1.4-fold, 1.6-fold, and 1.8-fold lower than the reference method, respectively, indicating relatively small inter-laboratory variation of the results using the kit.

The overall concordance of the data between ODK-1201 and the reference laboratory systems was acceptable (Fig. 1c), although the bias was slightly outside the stringent criteria previously suggested (Branford et al. 2008). After conversion to the IS, 74 % of values were within twofold, 94 % within threefold, and 98 % within fivefold of the reference laboratory value. The concordance in labelling a sample as MMR (i.e., a *BCR*-*ABL1* value of 0.10 % *BCR*-*ABL1*^IS^) between the reference method and the ODK-1201was determined as 84 % (N = 43/51) of samples.

**Fig. 2:**
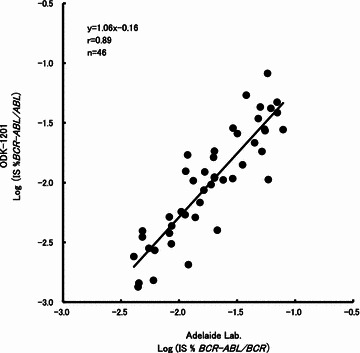
Correlation between the IS values obtained by ODK-1201 and the reference laboratory method in 46 clinical samples from CML patients with IS between lowest positive value (LPV) and 0.1 % *BCR*-*ABL1* ^IS^

Recent improvement in CML therapy with TKI requires detection of minimal residual disease with high sensitivity, i.e., less than 0.1 % *BCR*-*ABL1*^IS^. To verify the clinically-useful sensitivity of the ODK-1201 kit, we tested the correlation between the two methods in the assessment of 46 clinical samples with IS between the lowest positive value (LPV) and 0.1 % *BCR*-*ABL1*^IS^. A high correlation coefficient (r = 0.89) was found (Fig. 2), suggesting that ODK-1201 can detect transcripts at the level of 0.0032 % *BCR*-*ABL1*^IS^.

## Discussion

The development of molecular targeted drug therapy has changed the prognosis of patients with various cancers. One of the most successful models is chronic phase CML, which has a well-documented mechanism of carcinogenesis, the constitutively active BCR-ABL kinase generated by the Philadelphia chromosome translocation (Rowley 1973). TKIs target the oncoprotein and have dramatically improved the prognosis of patients with CML (Kantarjian et al. 2010; O’Brien et al. 2003; Saglio et al. 2010). It is important to accurately evaluate the therapeutic effect and appropriately modify the treatment for achieving optimal response. However, standardization of minimal residual disease quantification has been difficult to implement even in this model neoplasia. Here, we evaluated an RT-qPCR kit, ODK-1201, which can measure *BCR*-*ABL1* transcripts on the IS with high sensitivity, in a single tube, in two hours after the RNA extraction procedure.

The achievement of MMR has been recognized as the therapeutic goal for chronic-phase CML patients based on the results reported on the IRIS trial and subsequent ones (Baccarani et al. 2009; Hughes et al. 2003; Marin et al. 2008). Furthermore, molecular responses of less than 0.01 % *BCR*-*ABL1*^IS^ or deeper are becoming the next target. A few clinical trials have shown that approximately 40 % of chronic phase CML patients who achieved and sustained a deep molecular response could stop administration of TKI and maintain treatment free remission (Mahon et al. 2010; Ross et al. 2013). Thus, accurate and reproducible molecular monitoring is essential for treatment management in CML, and the lack of standardization may lead to a diagnostic misclassification. Nonetheless, it is not widely understood that the RT-qPCR technique potentially produces highly variable quantitative data which may affect the validity of results and decision on treatments (Zhang et al. 2007). Establishment of the laboratory-specific CF, which has been used for standardization based on the IS is lengthy, costly, and not realistic for many clinical laboratories, because of the limited number of reference laboratories, currently only Mannheim and Adelaide, with which to exchange samples (Cross et al. 2008). Furthermore, the CF is recommended to be revalidated on a yearly basis or in case of changing reagents for RT-qPCR at a local laboratory (Muller et al. 2010). To improve the harmonization process, a WHO international standard panel directly linked to the IS was developed in 2009 (White et al. 2010). However, the IS was initially designed to express the quantification of *BCR*-*ABL1* transcripts in the range of MMR (as 0.10 % *BCR*-*ABL1*^IS^) to standardized baseline (as 100 % *BCR*-*ABL1*^IS^) established in the IRIS study, but not deeper molecular response (Hughes et al. 2006). The WHO standard panel was validated only for *BCR*-*ABL1* detection in the range 0.01–10 % *BCR*-*ABL1*^IS^ (White et al. 2010). It means that validation of systems for measuring *BCR*-*ABL1* less than 0.01 % *BCR*-*ABL1*^IS^ has not been formally developed. Therefore, we carried out the current study to evaluate clinical usefulness of the ODK-1201, especially at the deep molecular response, by the clinical sample exchange, and showed that the kit detects *BCR*-*ABL1* at the level of 0.0032 % *BCR*-*ABL1*^IS^, as validated by a worldwide reference laboratory. The LPV of the kit is 0.0007 % *BCR*-*ABL1*^IS^ (Nakamae et al. 2015), suggesting that it has the potential to detect *BCR*-*ABL1* at the level of a 5-log reduction from the standard baseline (MR5). In contrast, when *BCR*-*ABL1*-negative samples were analyzed by the kit, *BCR*-*ABL1* transcripts was never detected, suggesting low false positivity of the kit (data not shown). Of note, the measurement can be carried out using only 7 mL of blood, a sample which is smaller than the previously recommended volume (Baccarani et al. 2013).

The variation of results obtained by RT-qPCR is due to each stage of the procedure, including sample collection, transportation time from clinics to laboratories, RNA extraction, reverse transcription, and the actual quantitative PCR (Branford et al. 2006). The difference in internal control genes also largely affects the variation. It is imperative to consider and optimize all parameters to obtain meaningful results reported on the IS. It is recommended that samples should be stabilized immediately after collection of blood from patients, to avoid RNA degradation. More importantly, each laboratory must carry out appropriate quality controls with every batch of samples in order to monitor for shifts in the data. It is essential to ensure that samples are re-tested if the quality control results fall outside the acceptable range (Branford et al. 2006). Of note, the mean bias between the local methods in the three local laboratories after IS conversion was relatively small, indicating accuracy and inter-laboratory reproducibility of the kit. All of the three locations are major commercial clinical laboratories in Japan with strict quality control, and two of them had experience in obtaining laboratory-specific CFs using their own procedure before participation in the current study (Yoshida et al. 2012). This may have led to the relatively small mean bias shown in the three laboratories using the kit. Further studies with the participation of several laboratories worldwide are warranted for advancing the standardization of RT-qPCR measuring *BCR*-*ABL1* using commercial kits.

## Conclusions

ODK-1201 is a rapid one-step high sensitivity RT-qPCR system for detecting *BCR*-*ABL1* based on the IS, which contributes a useful tool for evaluation of deep molecular responses and standardization of treatment in CML.

## Abbreviations

CML: chronic myeloid leukemia
RT-qPCR: real-time quantitative polymerase chain reaction
IS: international scale
TKI: tyrosine kinase inhibitors
MMR: major molecular response
IRIS: International Randomized Study of Interferon and STI571
CF: conversion factor
LPV: lowest positive value
